# Disparities in Stroke Patient-Reported Outcomes Measurement Between Healthcare Systems in Brazil

**DOI:** 10.3389/fneur.2022.857094

**Published:** 2022-05-06

**Authors:** Sheila Cristina Ouriques Martins, Wyllians Vendramini Borelli, Thais Leite Secchi, Gabriel Paulo Mantovani, Arthur Pille, Daissy Liliana Mora Cuervo, Leonardo Augusto Carbonera, Ana Claudia de Souza, Magda Carla Ouriques Martins, Rosane Brondani, Andrea Garcia de Almeida, Angélica Dal Pizzol, Franciele Pereira dos Santos, Ana Claudia Alves, Nathalia Soares Meier, Guilherme Pamplona Bueno Andrade, Pedro Angst Maciel, Alexandre Weber, Gustavo Dariva Machado, Mohamed Parrini, Luiz Antonio Nasi

**Affiliations:** ^1^Hospital Moinhos de Vento, Neurology Service and Postgraduate in Stroke Neurology, Porto Alegre, Brazil; ^2^Hospital de Clínicas de Porto Alegre, Neurology Service, Porto Alegre, Brazil; ^3^Faculty of Medicine, Universidade Federal do Rio Grande do Sul (UFRGS), Porto Alegre, Brazil; ^4^Brazilian Stroke Network, Porto Alegre, Brazil; ^5^Universidade Federal do Rio Grande do Sul, Pharmacology and Therapeutics Research Program, Porto Alegre, Brazil; ^6^Universidade Federal do Rio Grande do Sul, Postgraduate in Medical Sciences, Porto Alegre, Brazil; ^7^Hospital Moinhos de Vento, Porto Alegre, Brazil

**Keywords:** stroke, patient reported outcome (PROM), healthcare system, disparities health, stroke care, International Consortium for Health Outcomes Measurement, low- and middle-income countries

## Abstract

**Introduction:**

Acute stroke interventions, such as stroke units and reperfusion therapy, have the potential to improve outcomes. However, there are many disparities in patient characteristics and access to the best stroke care. Thus, we aim to compare patient-reported outcome measures (PROMs) after stroke in two stroke centers representing the public and private healthcare systems in Brazil.

**Methods:**

PROMs through the International Consortium for Health Outcomes Measures (ICHOM) were assessed at 90 days after the stroke to compare two Brazilian hospitals in southern Brazil: a public university and a private stroke center, both with stroke protocols and stroke units.

**Results:**

When compared with the private setting (*n* = 165), patients from the public hospital (*n* = 175) were younger, had poorer control of risk factors, had more frequent previous strokes, and arrived with more severe strokes. Both hospitals had a similar percentage of IV thrombolysis treatment. Only 5 patients received mechanical thrombectomy (MT), all in the private hospital. Public hospital patients presented significantly worse outcomes at 3 months, including worse quality of life and functional dependence (60 vs. 48%, *p* = 0.03). Poor outcome, as measured by the mRS score, was significantly associated with older age, higher NIHSS score, and the presence of heart failure. However, the public practice was a strong predictor of any self-reported disability.

**Conclusion:**

Patients assisted at a good quality public stroke center with the same protocol used in the private hospital presented worse disability as measured by mRS and patient-reported outcome measures, with greater inability to communicate, dress, toilet, feed, and walk.

## Introduction

Stroke has emerged as a major global health problem, and over 12 million people have a stroke each year and around 6.5 million people will die as a result (1). The burden is the greatest in low-and middle-income countries (LMIC), responsible for 85% of these deaths (1–3). In Latin America, stroke is the second leading cause of death (4), corresponding to 6.7% of the total deaths at a rate of 47.3 deaths per 100.000 inhabitants (5).

Disability in the post-stroke phase has a major impact on society. It is highly associated with functional dependence and the need to increase healthcare usage worldwide (6), besides its massive economic impact (6, 7). Individuals presenting with acute stroke may be severely affected by healthcare coverage, including the availability of ambulance services, stroke units, reperfusion therapy, or rehabilitation in different health settings. Disparities in healthcare systems also significantly impair recovery after stroke (8), with a substantial impact on LMIC. Despite the well-established benefit of acute interventions on stroke mortality and functional outcome, their implementation has been very slow, especially in the most vulnerable countries (9–11). A recent World Stroke Organization and World Health Organization study (9) evaluated 314 stroke centers in 84 countries and showed that only 35% of hospitals had the minimum structure to be considered a stroke center with the organization of acute stroke care and at least intravenous (IV) thrombolysis implemented. The availability of stroke units was 91% in high-income countries (HIC) and 18% in low-income countries (LIC), and acute stroke treatments were available 60% in HIC and upper-MIC and only 26% in LIC.

Brazil is a continental country with 213 million inhabitants. The public healthcare system, financed by the Ministry of Health, has universal and free healthcare coverage for the entire population (12, 13). The private system includes direct payment by the patient (<5% of cases) or by a private healthcare insurance (supported by companies or individually). Only 25% of the population has a private healthcare insurance (13). Brazil has huge disparities in healthcare access among the different regions and also, within the same region, between the public and private systems of care (10–12).

For 30 years, stroke was the leading cause of death in Brazil (12, 14). Stroke mortality has been decreasing in the last decade in the country (14, 15); however there are still 400.000 strokes and more than 100.000 deaths per year (12–14). Currently, 80% of the strokes are treated in public hospitals (12), but the organization of acute stroke care started in private hospitals in 2002 after the approval for the use of IV tissue plasminogen activator (tPA) in patients with acute ischemic stroke (AIS) by the National Regulatory Agency. Thereafter, in 2005, some public university hospitals implemented IV thrombolysis with local resources, but only in 2012 was published the National Stroke Policy, led by the Ministry of Health, that organized public hospitals as stroke centers with stroke units and a complete multidisciplinary stroke team, including the implementation of IV thrombolysis in the public healthcare system (12, 16, 17). Since then, the number of well-trained public and private stroke services has increased in Brazil.

Porto Alegre, a city in southern Brazil, was the first to implement a stroke network in the country, initially with 3 hospitals (2 public and 1 private) and currently with 15 public and 2 private stroke centers. Since 2005, Porto Alegre has had a very well-structured network for stroke care at all healthcare levels, from pre-hospital to hospital care (12). In 2015, Hospital Moinhos de Vento, an experienced private stroke center, began the implementation of Patient-Reported Outcome Measures (PROMs) to follow-up patients with stroke through the International Consortium for Health Outcomes Measurement (ICHOM) (18, 19). Hospital de Clínicas de Porto Alegre, the first public university hospital licensed by the Ministry of Health as a stroke center, started the same follow-up strategy in 2016. These two hospitals use the same stroke protocols and share several stroke team professionals.

PROMs are reliable indicators of quality of life after stroke (20). It comprises a set of questions directly related to the patient's functional outcomes, including the ability to perform activities independently without third-party interpretation. It has been used as an important tool for value-based healthcare, being a model of healthcare delivery in which providers, including hospitals and physicians, are paid based on the patient's health outcomes rather than paid by the services provided. However, functional outcomes measured with PROMs are still scarce in stroke care (21). In this context, Brazil is the ideal scenario to analyze the disparities related to public and private healthcare services, the analysis being done for the first time in the country, including PROMs.

Thus, in this study, we aim to compare the patient-reported outcomes measures from two well-structured stroke centers in Brazil that use the same stroke care protocols, but that represent distinct healthcare scenarios, public and private identifying which factors may predict outcomes for these patients.

## Materials and Methods

The study is based on a cohort of consecutive patients with acute ischemic stroke treated at 2 stroke centers in a city in southern Brazil: 1 private and 1 public university hospital. For this study, all consecutive patients with stroke assisted for 1 year at the private hospital (where the ICHOM strategy was implemented to measure PROMs since October 2015) and for 5 months at the public university hospital (where the ICHOM strategy was implemented from August to December 2016 as a feasibility pilot project to evaluate PROMs in stroke in a public setting) were evaluated. Importantly, both stroke centers used exactly the same stroke code protocol for individuals suspected of AIS.

### Study Settings

1. The Public University Hospital is a tertiary hospital with 850 beds, being a regional reference for acute stroke care in the Unified Public Health System (called SUS). It is a high-volume stroke center with ~600 patients with stroke per year, with an acute stroke unit inside the Emergency Department (ED) and a Stroke Rehabilitation Unit with a complete multidisciplinary team, including early in-patient rehabilitation for all patients. Since 2013, after the implementation of the National Stroke Policy, the number of public stroke centers has increased from 2 to 16 in the region. As a result of the aforementioned strategies, the number of thrombolysed patients has increased across the region, avoiding the burden on a single stroke center. Since then, the number of thrombolysed patients in this hospital is stable, between 65 and 80 patients per year. The stroke center staff is formed by a trained multidisciplinary stroke team, including 4 stroke neurologists who provide supervision and support to neurology residents onsite during the day and by telemedicine at night. Neurology residents provide onsite coverage during the day and on-call coverage from 6 pm to 8 am. So, the stroke team is available 24 h a day on a rotating schedule to give support for all stroke cases from arrival at the ED to hospital discharge. It is a Joint Commission International-accredited hospital with stroke outpatient clinic, where 80–85% of patients return for follow-up visits at least once after hospital discharge. Patients with lacunar stroke, transient ischemic attack, or minor stroke with complete etiological investigation during hospitalization are referred to primary care services. Rehabilitation after hospital discharge depends on the public healthcare system, and generally, the access is difficult with a long wait to start. In addition, the patient is often unable to pay for the transportation to go to the rehabilitation session.

2. The Private Hospital is a tertiary hospital with 465 beds, which only admits private patients or patients with private health insurance. It is affiliated with Johns Hopkins Medicine International and it is also a Joint Commission International accredited hospital. It has a Neurology Residency Program, a Neurohospitalist fellowship program, and a Latu Sensu Post-Graduate in stroke. It has a well-organized Stroke Center, with a service flow of approximately 250 patients with stroke per year and a complete multidisciplinary team. Usually, the hospital has 30 IV thrombolysis and 8 to 12 thrombectomies per year. After hospital discharge, patients are referred to their physician for outpatient care, so the 3-month outcome is collected over the phone by a quality-trained stroke coordinator. Hospital Moinhos de Vento was the first to implement the ICHOM methodology to measure outcomes in Brazil and consecutively monitor patients with stroke since October 2015. After hospital discharge, patients have quick rehabilitation support through private healthcare insurance.

### Data Collection and ICHOM Standard Set Outcomes

All data regarding baseline characteristics, pre-functional stroke status measured through modified Rankin Scale (mRS) score, risk factors, last time seen well, the severity of neurological deficit assessed by the National Institute of Health Stroke Scale (ranging from 0 to 42, higher values indicating worse outcomes), key quality metrics for acute stroke care, acute treatments, complications, and mRS score at hospital discharge are prospectively collected from all patients with stroke in the routine in both hospitals. Data collection is performed by residents, fellows, nurses, research coordinators, and trained medical students. The 3-month mRS is assessed in person at the outpatient clinic or by phone. The mRS score is a 7-level scale to evaluate functional outcome. It ranges from 0 to 6, with higher values indicating a worse outcome. In this study, poor functional outcome was defined as mRs > 2 (functional dependence).

Monitoring hospital performance indicators ensures adherence to guidelines, but it is not enough to understand what is important to patients. Therefore, in addition to the mRS, PROMs after stroke were evaluated through the International Consortium for Health Outcomes standard set (19). The ICHOM for stroke was developed in 2015 by an international panel of experts who have consensually established a set of global standards to measure outcomes that matter the most for patients with stroke. It aims to achieve comparable results and help organize healthcare services by engaging patients, healthcare providers, and health system funders to improve outcomes and reduce costs (18).

The ICHOM standard set (19) outcomes at 90 days after the index event were assessed with a 20-topic questionnaire tracked using the Patient-Reported Outcomes Measurement Information System short questionnaire (PROMIS-10), the Modified Rankin Scale Questionnaire, overall survival, stroke recurrence, smoke cessation, and 5 more questions about post-stroke functional status (walking, toileting, dressing, eating, and communication skills).

The PROMIS-10 is a 10-item instrument that assesses the general domains of health and functioning, including general physical health, mental health, social health, pain, fatigue, and overall perceived quality of life. The total is the global score, but it can also be scored on the Global Physical Health component (0–20 points) and Global Mental Health component (0–20 points). Higher scores reflect better performance. The EuroQol group index score (EQ-5D) was calculated using a transformation formula (19). This scale ranges from −0.33 to 1, where higher values indicate a better quality of life.

### Statistical Analysis

Frequencies and categorical variables were compared using Chi-squared tests. Continuous variables were presented as median (interquartile range-IQR), except for functional outcomes measures scores (EQ-5D and PROMIS-10). When appropriate, group comparisons between the two care settings were performed using parametric and non-parametric tests. A multivariate regression model was performed to evaluate the predictors of poor outcome. The variables in the model were based on results from other studies or clinical observation. Variables with *p* < 0.05 in the univariate comparisons between care settings (public vs. private) at baseline were also included. One model evaluated the predictors of any type of disability and another regression model for modified Rankin scale score as a continuous variable. We performed Bonferroni's correction for multiple comparisons and *p*-values were considered significant at <0.05. Data analysis was performed using R 3.6.2 (R foundation for statistical computing, 2016), and variables with missing values above 9% were excluded.

### Ethics

The study protocol and cohorts from both hospitals were approved by the Ethics Committee. The consent form was waived by the ethics committees because the data are collected as part of the monitoring the quality of care and with the objective of guiding better the recommendations for post-stroke care. Patient personal data is not shared under any circumstances.

## Results

**Figure 1:**
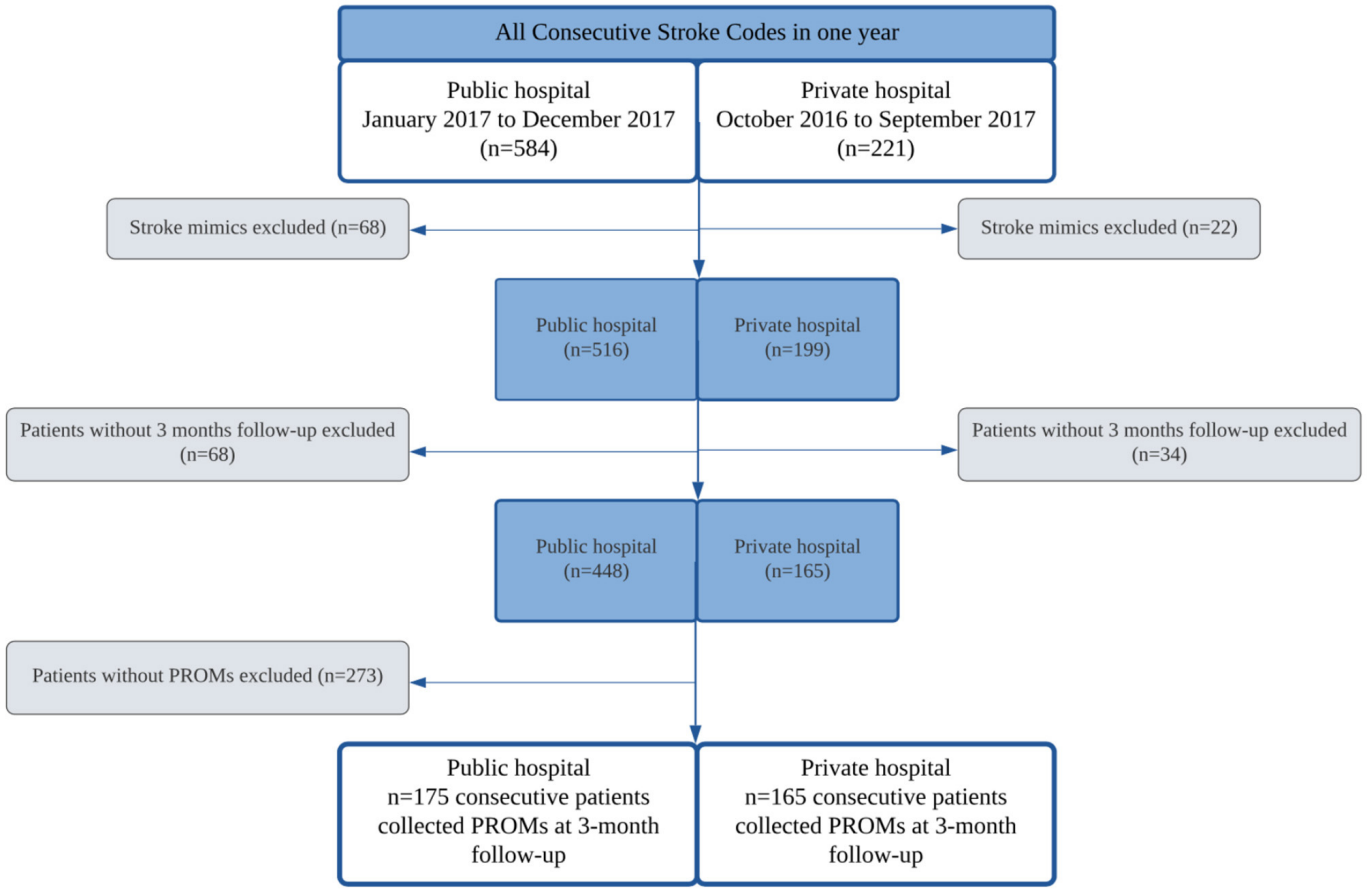
Flowchart of the patients included in the study.

The flowchart of patients included in the study is shown in Figure 1. Of all consecutive patients assisted for acute stroke during 1 year, all patients with patient-reported outcomes collected at 3 months at both hospitals were included in the study. PROMs were collected for all patients consecutively for 1 year in the private hospital and for 5 months in a pilot for the feasibility of implementation in the public hospital. During the study period, 82% of patients were evaluated for a 3-month follow-up at the public hospital and 83% at the private hospital. The sample consisted of 340 individuals (142 females, 41.76%), included in both public (*n* = 175) and private (*n* = 165) tertiary hospitals. Individuals from the public system were younger [68 (59–77) vs. 77 (67–85), *p* < 0.0001], showed a higher frequency of hypertension (82 vs. 67%, *p* = 0.002), diabetes (36 vs. 25%, *p* = 0.04), heart failure (11 vs. 4%, *p* = 0.03), history of drinking and smoking (6 vs. 3% and 41 vs. 18%, respectively), previous history of stroke (27 vs. 16%, *p* = 0.02), and more severe neurological deficit on arrival [median NIHSS score 5 (3–11) vs. 3.5 (0–7), *p* = 0.01] (Table 1). Individuals arrived at the emergency department in a median of 256 min [118–520] after symptom onset, without difference between the hospitals, with 48% in the public and 55% in private arriving in <4.5 h (*p* = 0.28). In addition, both hospitals had a similar percentage of ischemic patients with stroke treated with IV thrombolysis (16% in the public hospital vs. 23% in the private hospital, *p* = 0.19), but the door-to-needle time was lower in the private hospital [43 min (35–61) vs. 81 min (63–110), *p* = 0.0001]. Only 5 patients received mechanical thrombectomy, all in the private hospital (MT was not available at the public hospital during the study period).

**Table 1:** Baseline characteristics.

	**Public hospital (*n* = 175)**	**Private hospital** **(*n* = 165)**	***p*-value**
Age, median (IQR)	68 [59–77]	77 [967–85]	**<0.0001**
Female Sex, *n* (%)	74 (42)	68 (41)	0.91
NIHSS at admission, median (IQR)	5 [3–11]	3.5 [0–7]	**0.01**
Time from symptom onset to admission, median (IQR)	287 [122–529]	218 [107–506]	0.25
Arrival within 4.5 h, *n* (%)	82 (48)	78 (55)	0.26
Door to needle time, median (IQR)	81 [63–110]	43 (35–42)	**0.0001**
**Stroke type**			**0.003**
Ischemic or Transient ischemic attack	158 (90)	144 (87)	
Ischemic stroke, *n* (%)	147 (84)	124 (75)	
Transient ischemic attack, *n* (%)	11 (6)	20 (12)	
Hemorrhagic stroke, *n* (%)	11 (6)	21 (13)	
Others, *n* (%)	6 (3)	0	
Alteplase administration (among ischemic stroke), *n* (%)	23 (16)	28 (23)	0.19
Mechanical thrombectomy (among ischemic stroke), *n* (%)	0	5 (4)	NA
**Risk factors**			
Hypertension, *n* (%)	141 (82.5%)	109 (67.3%)	**0.002**
Diabetes, *n* (%)	61 (35.7%)	41 (25.3%)	**0.04**
Dyslipidemia, *n* (%)	20 (11.7%)	28 (17.4%)	0.16
Current or previous drinking, *n* (%)	10 (5.9%)	5 (3.1%)	**0.02**
Current or previous smoking, *n* (%)	70 (40.9%)	29 (17.9%)	**<0.0001**
Obesity, *n* (%)	9 (6.5%)	13 (8.1%)	0.66
Ischemic cardiopathy, *n* (%)	28 (16.7%)	16 (10.0%)	0.10
Myocardial infarction, *n* (%)	7 (5.1%)	4 (2.5%)	0.36
Heart failure, *n* (%)	18 (10.7%)	7 (4.4%)	**0.03**
Atrial fibrillation, *n* (%)	31 (18.3%)	30 (18.8%)	1.0
Previous stroke, *n* (%)	45 (27.3%)	26 (16.4%)	**0.02**
Prestroke score on the modified Rankin scale > 1	24 (13.7%)	18 (10.9%)	0.43

*The bold values indicate the difference between groups that are statistically significant*.

Overall mortality was similar between both hospitals (*p* > 0.05, Table 2). However, patients from the public institution had significantly more disabilities at 3-month follow-up when compared with patients from the private hospital. The mean of the mRS was higher in patients from the public setting (3.2 ± 2.1 vs. 2.6 ± 2.3, *p* = 0.005), either by the proportion of patients with some disability measured as mRS > 1 (79 vs. 59%, *p* < 0.0001) or by functional dependence defined as mRS > 2 (60 vs. 48%, *p* = 0.03).

**Table 2:** 3-month outcome measurement accord to private and public hospital.

	**Public hospital (*n* = 175)**	**Private hospital (*n* = 165)**	***p*-value**
Overall mortality	39 (22%)	31 (19%)	0.50
mRS, mean (SD)	3.3 (±2.1)	2.6 (±2.3)	**0.005**
***mRS (>1)***	138 (79%)	97 (59%)	**<0.0001**
Ischemic stroke with thrombolysis	17 / 21 (80%)	16 / 28 (57%)	0.23
Ischemic stroke without thrombolysis	100 / 124 (81%)	59 / 95 (62%)	**0.003**
Hemorrhagic stroke	11 / 11 (100%)	17 / 21 (80%)	0.32
***mRS (>2*** **)**	104 (60%)	78 (48%)	**0.03**
Ischemic stroke with thrombolysis	12 / 22 (55%)	15 / 28 (54%)	1
Ischemic stroke without thrombolysis	76 / 124 (61%)	46 / 95 (48%)	0.07
Hemorrhagic stroke	9 / 11 (82%)	15 / 21 (71%)	0.83
Rehabilitation after discharge	61/99 (46%)	23/60 (38%)	0.43
Physical therapy after discharge (for *mRs > 1*)	53 / 98 (54%)	15 / 21 (71.4%)	0.22
**3-month ICHOM outcomes**			
EQ5D, mean (SD)	0.58 (±0.07)	0.61 (±0.06)	0.001
**PROMIS-10 scores**			
Global score, mean (SD)	22.5 (±3.6)	24.7 (±3.5)	**<0.0001**
Physical Health score, mean (SD)	11.2 (±1.9)	11.8 (±1.6)	**0.002**
Mental Health Score, mean (SD)	11.3 (±3.5)	12.9 (±3.1)	**0.0003**
**PROMIS-10 individual items**			
General Health status (excellent/very good)	20 (15%)	38 (30%)	**0.005**
Quality of life (excellent/very good)	18 (13%)	43 (34%)	**0.0006**
Physical health (excellent/very good)	11 (8%)	31 (24%)	**0.0002**
Global mood/cognition (excellent/very good)	30 (22%)	49 (38%)	**<0.0001**
Social participation (excellent/very good)	40 (30%)	45 (35%)	**0.003**
Carry out social activities/roles (excellent/very good)	51 (38%)	36 (28%)	0.32
Daily physical activities (completely or mostly)	67 (50%)	96 (75%)	**<0.0001**
Mood problems (never/almost never)	26 (19%)	13 (10%)	0.053
Fatigue (none/mild)	73 (54%)	83 (64%)	**0.0007**
Pain (none/mild)	46 (34%)	55 (43%)	**<0.0001**
Walking without help	87 (64%)	102 (79%)	**0.011**
Feeding without tube	126 (93%)	122 (96%)	0.15
Able to communicate	78 (58%)	106 (84%)	**<0.0001**
Toileting independently	95 (70%)	104 (81%)	**0.045**
Dress independently	85 (63%)	104 (81%)	**0.001**
Self-reported new stroke	33 (19%)	4 (2%)	**<0.0001**
Stopped smoking (among previously smokers)	28 / 36 (78%)	8 / 12 (67%)	0.7

*ICHOM, International Consortium for Health Outcomes. The bold values indicate the difference between groups that are statistically significant*.

**Figure 2:**
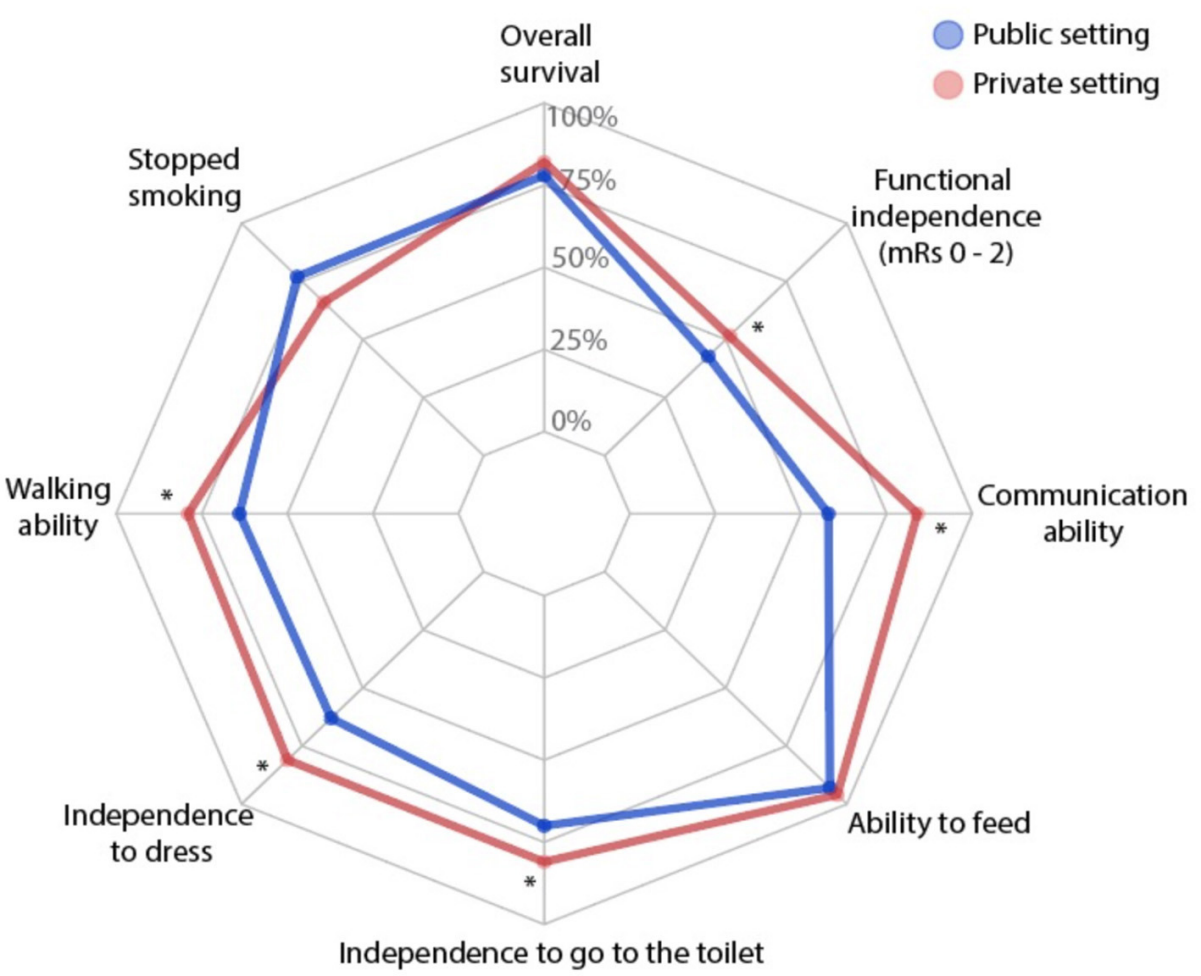
Patient-reported outcomes measurement of post-stroke patients in a public (blue) and a private (red) settings. The external line represents 100% of patients with good outcomes in each domain. *p < 0.05.

Patient-reported outcome measures were collected at a 3-month follow-up and the results are presented in Table 2. The Global PROMIS-10 score was worse in patients from the public setting (*p* < 0.0001), accounting for the physical and mental health subscores of the test (*p* < 0.01 for both). The proportion of good results for almost all PROMIS-10 items was lower in patients from the public hospital (Table 2). Also, the quality of life evaluated through the EQ5D score was worse in public healthcare. These patients had a decreased rate of self-reported ability to walk, communicate, toilet, and dress independently (Table 2, Figure 2). Figure 2 clearly shows the worst outcome in the public hospital in almost all functions evaluated, with the outer line representing 100% of patients with a good outcome in each domain. As this study showed PROMs from consecutive patients assisted for 5 months at the public hospital and 1 year at the private, we performed a 90-day mRS analysis of all acute strokes over 1 year at the public hospital and compared it with mRS scores during 5 months presented in our study to assess whether the results are representative of hospital patients. Of the 540 patients assisted during 1 year at the public hospital, 90% had mRS score at 90 days of follow-up. Of these, 74.3% had mRS > 1 compared with 79% of the 175 patients assisted during the 5 months presented in this study (*p* = 0.26).

Despite worse outcomes and higher rates of disability in the public setting, individuals with mRS > 1 received more physical therapy in the private practice when compared with public (71 vs. 54%), although it did not reach statistical significance. The post-discharge rehabilitation assessment started in May 2016, so only 36% of the private hospital patients were evaluated. The proportion of patients with stroke recurrence at 3-month follow-up was higher in the public hospital (19 vs. 2%, *p* < 0.0001).

In a multivariate regression model (Table 3), age, NIHSS score, hypertension, diabetes, heart failure, atrial fibrillation, smoking, alcohol abuse, previous stroke, pre-stroke mRS, door-to-needle time, thrombolysis, and hospital were included. “At 3-months, poor outcome as measured by mRS score was significantly associated with older age, higher NIHSS score, and the presence of heart failure. However, public practice was a strong predictor of any self-reported disability, in addition to the presence of hypertension and higher NIHSS score at arrival. Thrombolysis was a protector, reducing the risk of poor outcome by 76%.”

**Table 3:** Multivariate regression model showing independent predictors of poor outcome at 3-months.

**Variable**	**Estimate**	***t*-value**	***p*-value**	**OR (95% CI)**
**A) 3-month mRs**				
Age	0.01	2.11	0.03	1.01 (1–1.03)
NIHSS at arrival	0.13	7.67	<0.0001	1.14 (1.1–1.7)
Stroke type - TIA	−1.01	−2.62	0.009	0.36 (0.17–0.77)
Heart failure	1.03	2.4	0.01	2.79 (1.2–6.5)
**B) Any self-reported disability**				
Hypertension	−1.82	2.55	0.0006	3.52 (1.39–9.9)
NIHSS at arrival	0.27	5.57	<0.0001	1.31 (1.2–1.45)
Thrombolysis administration	−1.4	−2.5	0.01	0.24 (0.07–0.7)
Private practice	−1.1	−2.97	0.003	0.3 (0.15–0.67)

## Discussion

This study demonstrated disparities between patients with acute stroke treated at two Brazilian hospitals from different healthcare systems. The sample described is composed of adults from a middle-income country with a mixed healthcare system, which is a proxy of both high and low-income countrie's health systems. Importantly, sampling was performed identically between centers, and both hospitals had the same neurologist team and the same protocols. Thus, the results of this study reflect distinctions particularly related to the healthcare system, including patient profiles and outcomes. Although patients from the public hospital were younger (68 vs. 77 years old), they had more risk factors, with nearly 27% having a prior stroke compared with 16% in the private hospital, and they had more severe strokes. The two hospitals had good rates of IV thrombolysis, and nearly half of the patients arrived within 4.5 h of symptoms at both hospitals. The main findings of this study showed worse results on almost all disability and quality of life outcome measures in the public hospital: worse functional outcome measure by mRS; more patients with a disability to walk, communicate, toilet, and dress independently; and worse scores on quality of life scales, including mood, cognitive function, pain, and fatigue. Furthermore, the proportion of patients with a new stroke was much higher in the public hospital (19 vs. 2%). Interpretations should be made with caution, although they have the potential to reflect the discrepancy between high and low-health settings worldwide.

Patients with stroke in resource-limited settings are generally younger (22–25) and have more uncontrolled risk factors, in addition to having less access to prevention (26) and less knowledge about stroke (27–29). This context contributes to a higher incidence and recurrence of stroke in LMIC and also to more severe strokes. In our study, patients from the public system had more cardiovascular risk factors, more severe strokes, and had worse outcomes than those from the private setting. However, the only comorbidity independently associated with poorer mRS, after regression analysis, was heart failure, which suggests that other factors play important roles in stroke functional outcomes, such as social determinants of health (30). Individuals assisted by the public healthcare system tend to come from more vulnerable socio-demographic contexts, with lesser availability of medical assistance and worse health conditions than the holders of private health plans (31). Social determinants are essential for providing adequate health care (32), and the socioeconomic context of families living in poverty is a well-known predictor factor of poor recovery in individuals with stroke, where some patients may not even be able to take a bus to go to physical therapy (33).

Despite similar mortality rates, patients from public and private settings presented divergent functional outcomes. The two hospitals had similarly good rates of IV thrombolysis, showing that they are organized for acute stroke care; however patient-reported outcomes were significantly worse in patients from the public setting in all categories evaluated except for the use of a feeding tube. Importantly, almost half of the patients in the public setting showed an inability to communicate in the 3-month follow-up, in contrast to the private hospital, where it was only 16%. Besides, a large number of patients remained with important limitations in motor function after 3 months of stroke, including the ability to walk, toilet, and dress independently, in addition to the greater severity of stroke at the hospital arrival, which may reflect that these patients are receiving insufficient rehabilitation after hospital discharge, both physical and speech therapy, which plays an important role in the stroke outcome (34). Although not statistically significant, post-discharge physiotherapy was more present in the private hospital, although patients from the public hospital needed more. This is in agreement with previous evidence, which indicates that the majority of physiotherapists in Brazil work in private practice (35), while only a minority of the Brazilian population has health insurance (12, 31). In our study, both hospitals had a good rehabilitation structure, but access after hospital discharge was worse in the public hospital.

Public health strategies aimed at predictors of functional outcomes may massively improve the quality of care in public and private practice. Evidence-based policies have dramatically improved acute stroke care in Latin America with important advances in stroke prevention, yet to be implemented worldwide (10, 11). Hypertension is a very common risk factor for a myriad of health conditions, potentially manageable in primary care. Thrombolytic therapy, a well-known predictor of better stroke outcomes (36), decreased the risk of poor outcome by 76% in our study. Mechanical thrombectomy (MT) was used in only 5 cases in the private hospital. In the public hospital, MT was not available in 2016. It was approved in 2021 after the RESILIENT Study (37), a Ministry of Health clinical trial in the Brazilian public healthcare system. As a result, there will be an increase in the availability of reperfusion therapies, including MT in public hospitals, which will possibly decrease disability after stroke.

Private practice was a protective factor for any type of self-reported disability. In addition to the lower number of risk factors and lower stroke severity, potential explanations include the presence of MT only in the private hospital and better assistance after hospital discharge (38). Individuals with private health insurance also had better socioeconomic conditions (31). Importantly, a previous study showed that the stroke program may standardize the quality of care in hospitals with different resources (39). In both hospitals in this study, stroke care management was standardized and the only treatment not available in the public hospital was MT, currently approved in the public healthcare system in Brazil. In low-resource settings, the availability of stroke services is lower worldwide (9), and there is also a huge gap in access to primary and secondary prevention and post-discharge rehabilitation (9, 10).

Some limitations of this study should be taken into consideration. We selected individuals from a large public university hospital with more resources than the majority of tertiary centers in Brazil. Thus, our results may overestimate the functional outcomes in the public system across the country and, probably, in other hospitals the disparities would be greater. In both settings, stroke mortality was higher than expected, with no difference between hospitals (20 vs. 18%). This result probably occurred because all deaths were accounted for in the study, including reviewing the medical records, in addition to the fact that only 82% of patients from both services were evaluated. The strength of this study is the use of PROMs to compare stroke outcomes in both healthcare systems in Brazil. Despite a growing interest in measuring outcomes that matter to patients, there are still few randomized clinical trials evaluating PROMs, especially rehabilitation-related studies (21, 40). PROMs can help to better address the needs of patients after a stroke, being more sensitive to the detection of disability. PROMs can detect pain, fatigue, depression, as well as changes in diet, speech, and walking. Thus, through this measure, it is known exactly which deficit has the greatest impact on life after a stroke, allowing for a more specific guidance of a rehabilitation program and public health strategies (21, 41).

In summary, patients from a good-quality public stroke center presented worse patient-reported outcome measures at follow-up, namely, the ability to walk, communicate, toilet, dress, and the quality of life. These results occurred despite similar rates of IV thrombolysis, which was an independent predictor of outcome, reducing poor outcome by 76%. Furthermore, they had significantly worse control of cardiovascular risk factors and increased stroke recurrence, but similar mortality after 3 months of follow-up when compared with patients from the private setting. In the last decade, many improvements have occurred in the organization of acute stroke care in Brazil, with the increase in the number and quality of stroke centers. However, the country still has many young patients who arrive with severe strokes, with several risk factors and poor access to rehabilitation after hospital discharge. The country needs to continue increasing the number of stroke centers, but the main strategy should be a strong national primary care program, with more access to primary and secondary prevention and rehabilitation.

## Data Availability Statement

The raw data including the R Script will be made available by the authors, without undue reservation.

## Ethics Statement

The studies involving human participants were reviewed and approved by Comitê de Ética em Pesquisa Hospital Moinhos de Vento and Comitê de Ética em Pesquisa Hospital de Clínicas de Porto Alegre. Written informed consent for participation was not required for this study in accordance with the national legislation and the institutional requirements.

## Author Contributions

SM analyzed the data, prepared the first draft of the manuscript, and reviewed all drafts. WB performed the statistical analysis and prepared the first draft of the manuscript. TS provided data, reviewed and edited the first draft, and final version of the manuscript. GMan prepared the first draft of the manuscript and reviewed the final version. AP edited the first draft and reviewed the final version of the manuscript. DM, LC, AS, MM, RB, AAlm, AD, FS, AAlv, NM, GA, PM, AW, GMac, MP, and LN provided data, reviewed the result, and approved the final version of the manuscript. All authors contributed to the article and approved the submitted version.

## Funding

The publication of this study was funded by FIPE/HCPA.

## Conflict of Interest

The authors declare that the research was conducted in the absence of any commercial or financial relationships that could be construed as a potential conflict of interest.

## Publisher's Note

All claims expressed in this article are solely those of the authors and do not necessarily represent those of their affiliated organizations, or those of the publisher, the editors and the reviewers. Any product that may be evaluated in this article, or claim that may be made by its manufacturer, is not guaranteed or endorsed by the publisher.
